# Pediatric Coping During Venipuncture With Virtual Reality: Pilot Randomized Controlled Trial

**DOI:** 10.2196/26040

**Published:** 2021-07-28

**Authors:** Therese Canares, Carisa Parrish, Christine Santos, Alia Badawi, Alyssa Stewart, Keith Kleinman, Kevin Psoter, Joseph McGuire

**Affiliations:** 1 Division of Pediatric Emergency Medicine Department of Pediatrics Johns Hopkins University School of Medicine Baltimore, MD United States; 2 Division of Child and Adolescent Psychiatry Johns Hopkins University School of Medicine Baltimore, MD United States; 3 Department of Child Life Johns Hopkins University School of Medicine Baltimore, MD United States; 4 Division of General Pediatrics Department of Pediatrics Johns Hopkins University School of Medicine Baltimore, MD United States

**Keywords:** pediatrics, psychological distress, virtual reality, procedural pain, anxiety, phlebotomy

## Abstract

**Background:**

Virtual reality (VR) has shown promise in reducing children’s pain and anxiety during venipuncture, but studies on VR lack objective observations of pediatric coping. Notably, the process of capturing objective behavioral coping data can be labor- and personnel-intensive.

**Objective:**

The primary aims of this pilot trial were to assess the feasibility of conducting a trial of VR in a pediatric emergency department and the feasibility of documenting observed coping behaviors during pediatric procedures. Secondarily, this study examined whether VR affects child and caregiver coping and distress during venipuncture in the pediatric emergency department.

**Methods:**

This stratified, randomized, controlled pilot trial compared coping and distress between child life–supported VR engagement and child life specialist support without VR during painful procedures in children aged 7-22 years in the pediatric emergency department. An external control (reference group) received no standardized support. Primary feasibility outcomes included rates of recruitment, rates of withdrawal from VR, and rates of completed Child Adult Medical Procedure Interaction Scale-Short Form (CAMPIS-SF) observations. Secondary clinical outcomes were applied to venipuncture procedures and included CAMPIS-SF coping and distress (range 0-1.0), pain and anxiety on a visual analog scale (range 0-10), and cybersickness symptoms.

**Results:**

Overall recruitment was 93% (66/71), VR withdrawal rate was 27% (4/15), and of the completed procedures, 100% (63/63) CAMPIS-SF observations were completed. A total of 55 patients undergoing venipuncture in the pediatric emergency department were included in the analyses of clinical outcomes: 15 patients (15 caregivers) randomized to VR, 20 patients (15 caregivers) randomized to child life specialist support, and 20 patients (17 caregivers) in the reference group. Patient coping differed across groups with higher coping in the VR group and child life specialist group than in the reference group (*P*=.046). There were no significant differences in the distress and pain ratings for patients and caregivers between the groups. Caregivers rated the lowest perceived anxiety in the child life specialist group (*P*=.03). There was no apparent change in cybersickness symptoms before and after VR use (*P*=.37).

**Conclusions:**

Real-time documentation of observed behaviors in patients and caregivers was feasible during medical procedures in which VR was utilized, particularly with the availability of research staff. VR and child life specialists improved coping in children during venipuncture procedures. Given the high participation rate, future studies to evaluate the efficacy of VR are recommended to determine whether an off-the-shelf VR headset can be a low-cost and low-risk tool to improve children’s coping during venipuncture or other related procedures.

**Trial Registration:**

ClinicalTrials.gov NCT03686176; https://clinicaltrials.gov/ct2/show/NCT03686176

## Introduction

Venipuncture is a common pediatric emergency department procedure; yet, optimal psychological interventions to promote coping remain undetermined. Standard of care ranges from no intervention to certified child life specialist support with use of a variety of cognitive or behavioral strategies. Data on virtual reality (VR) have overall demonstrated improved pediatric pain and anxiety during venipuncture [1-4], although 1 study found no change in pain [5]. These studies evaluated VR games that cater to a medical procedure (eg, a field of view that minimizes head movement or interaction that does not require a hand controller) [1-3,6]. Customized VR games for medical procedures are either designed locally and not available for dissemination or require a costly subscription. No prior studies have evaluated an off-the-shelf commercially available VR headset during pediatric procedures. Furthermore, prior VR studies evaluated outcomes of self-reported pain and anxiety, which are subjective and less meaningful in younger children. To our knowledge, no study to date has reported objective observations of children and caregiver’s coping behaviors. Observational measures of coping behaviors offer several advantages, including objectivity, inclusion of all age ranges, and inclusion of caregiver behaviors [7]. Since a caregiver’s response influences children’s coping [8], understanding caregiver behaviors may also elucidate the potential benefits of VR during pediatric procedures. While objective observations of child and caregiver behaviors are informative, the process of capturing these data can be labor- and personnel-intensive.

We conducted a pilot study to understand the feasibility of conducting a trial with a commercially available VR headset in a pediatric emergency department and the feasibility of documenting observed coping behaviors during pediatric procedures. Secondarily, this study examined whether VR affects child and caregiver coping and distress during venipuncture in the pediatric emergency department. The results of this study provide preliminary data for the planning of subsequent studies.

## Methods

### Study Design, Setting, and Sampling Technique

A convenience sample of patients aged 7-22 years who required a painful procedure (eg, venipuncture, laceration repair, burn debridement) in the pediatric emergency department were recruited. The study design was a stratified, randomized, controlled pilot trial that compared coping and distress between child life specialist–supported VR engagement and child life specialist support (clinicaltrials.gov NCT03686176). This study was conducted in an academic, urban, tertiary care pediatric emergency department. Randomization allocation was 1:1, performed in randomized blocks of 2, 4, 6, and 8 (R, Version 3.2.2, 2018), and stratified by the type of procedure. The block randomization allocation was imported into REDCap (version 10.0.28, 2019) [9,10] and performed by research assistants upon recruitment. Consistent with the recommendations by Kraemer et al [11], this pilot trial will be used to refine the research protocol, establish the infrastructure, address pragmatic issues, and gather pilot data to answer key questions about the use of VR during pediatric procedures. An external control (reference) group was enrolled when VR and child life specialists were unavailable.

### Inclusion and Exclusion Criteria

Inclusion criteria were patients aged 7-22 years who were in the pediatric emergency department and were undergoing any of the following procedures: burn debridement or dressing change, laceration repair, venipuncture (intravenous line or blood draw), abscess incision and drainage, fracture reduction or cast placement, or implanted central venous port placement. Exclusion criteria included severe developmental delays, seizures, blindness, trauma/infection on the head/face, altered mental status, medical urgency, and non-English speakers. Caregivers provided verbal consent and patients provided verbal assent to participate in the study.

### Study Protocol

Patient eligibility was screened by research assistants. Eligible patients and caregivers were introduced to the study by research assistants. Research assistants discussed the aims, risks, and benefits of the study, described the VR intervention, and invited patients and caregivers to participate. If consent was obtained, patients were block randomized as described above. Patients randomized to VR played a game using a commercially available VR headset with child life specialist support (*Oculus Go*, version 6.0. Facebook Technologies, 2018). Prior to the study start, child life specialists selected and downloaded VR games and apps, including a variety of passive VR experiences and active game play. Specific VR games/apps included *Netflix* version 1.1, *Bait!* version 1.11.61278 (Resolution Games; 2016), *Epic Roller Coasters* version 6.22.0 (Balneário Camboriú), BR: B4T Games; 2017, *Temple Run* version 1.0.4 (Imangi Studios, 2015), and *Disney Movies VR* version 1.6.472 (Walt Disney Studios, 2017). For all patients in the child life specialist support and VR groups, child life specialists performed a psychosocial assessment that considered child, family, and health care variables to determine how to support the patient during the procedure. For the VR group, child life specialists offered simple descriptions of select developmentally appropriate VR experiences, thereby allowing the child to then make a choice of VR games based upon their personal interests. Prior to VR use, the device was cleaned with hospital-grade disinfectant wipes. A disposable paper face shield and a disposable surgical cap were used for infection control. VR play was limited to 30 minutes to minimize development of cybersickness symptoms. The duration of VR play was monitored by child life specialists and the research assistant. The duration of the procedure served as a surrogate for VR duration, as the VR headset was applied immediately before the procedure and VR play completed after the procedure. Patients did not pretrain on VR games/application prior to their procedure start. Patients were empowered to discontinue VR at any point during the procedure upon verbal request or by removing the headset. Patients explored and experienced the VR game/application independently during their procedure and could request technical support from child life specialists when needed. Child life specialists provided technical support, including headset fit, menu navigation, or selection of an alternate game/application. Patients immersed in VR could opt for a tactile or verbal prompt just prior to the painful part of the procedure.

The active control group received child life specialist support and distraction of the child’s choosing. The reference group received no standardized support. During the procedures, an independent evaluator (ie, research assistant) logged the frequency of patient coping/distress and caregiver coping-promoting/distress-promoting behaviors in 1-minute increments by using a validated scale (Child Adult Medical Procedure Interaction Scale-Short Form [CAMPIS-SF], Multimedia Appendix 1) [12]. The work describing adult and child coping and distress behaviors was originally described by Blount et al [13]. CAMPIS-SF was selected because it is an abbreviated version of CAMPIS and is a validated and objective measure of coping and distress that has been studied in school-aged and adolescent-aged children [12,14,15]. Each independent evaluator was trained by the lead investigator (TC) to code behavior observations using the CAMPIS-SF scale. During training, the evaluators reviewed and coded prerecorded videos of children undergoing intravenous placement. They continued coding until interrater reliability (κ) was ≥0.8. In real time, during procedures, independent evaluators recorded behavior events of CAMPIS-SF on a paper scoring sheet. No video recordings were taken during this study. They utilized a 1-minute timer that provided a visual and audio cue to each minute interval. They later transcribed the CAMPIS-SF scores and other demographic or clinical data into REDCap.

### Feasibility Outcomes

The primary (feasibility) outcomes were the recruitment rate, defined as the number of patients who enrolled divided by those invited to participate; the withdrawal rate of VR, defined as the number of patients who stopped VR engagement divided by the number of patients who completed a procedure and were randomized to VR; and completion percentage of CAMPIS-SF observations for each patient/caregiver dyad of completed procedures. Feasibility benchmarks for each outcome were set at 80% or higher, and the target sample size for the feasibility outcomes of this pilot trial was at least 12 patients in each arm [16]. This pilot study will inform us of the feasibility to perform a full randomized controlled trial that is powered to detect changes in patients’ coping and distress (CAMPIS-SF) scores.

### Clinical Outcomes

The secondary (clinical) outcomes were CAMPIS-SF coping and distress scores. Coping scores were calculated by summing all coping events divided by the sum of all the coded behaviors exhibited during the procedure and reported as a proportion of the total behaviors (range 0-1.0). Distress scores were calculated similarly—the sum of all distress events divided by the sum of all behavior events. Secondary clinical outcomes also included change in pain and anxiety on a 10-point visual analog scale from baseline to peak levels during the procedure (range –10 to 10) and cybersickness symptoms [17]. Research assistants showed patients the visual analog scale and asked them to select a number (0-10) that characterized their pain and anxiety. Patients reported a pain or anxiety score before the procedure, and after the procedure, they were asked to report their peak pain or anxiety score experienced during the procedure. The change in pain or anxiety was calculated as postprocedure minus preprocedure. Owing to limited enrollment of other procedures, this study reported clinical outcomes on the subgroup of venipuncture (blood draw or intravenous line).

### Statistical Analysis

Feasibility outcomes were summarized. Demographics and clinical characteristics and outcomes were compared across groups by using analysis of variance for continuous variables and Fisher exact tests for categorical variables. A *P* value less than .05 was considered statistically significant. Patients whose procedures were not performed (eg, cancelled) or proceeded without study personnel present owing to medical urgency were excluded from the analysis. Analysis was performed with the intention-to-treat model.

### Ethical Considerations

This study was approved by the Johns Hopkins University School of Medicine institutional review board (ID IRB00161331).

## Results

### Patient Enrollment

**Figure 1:**
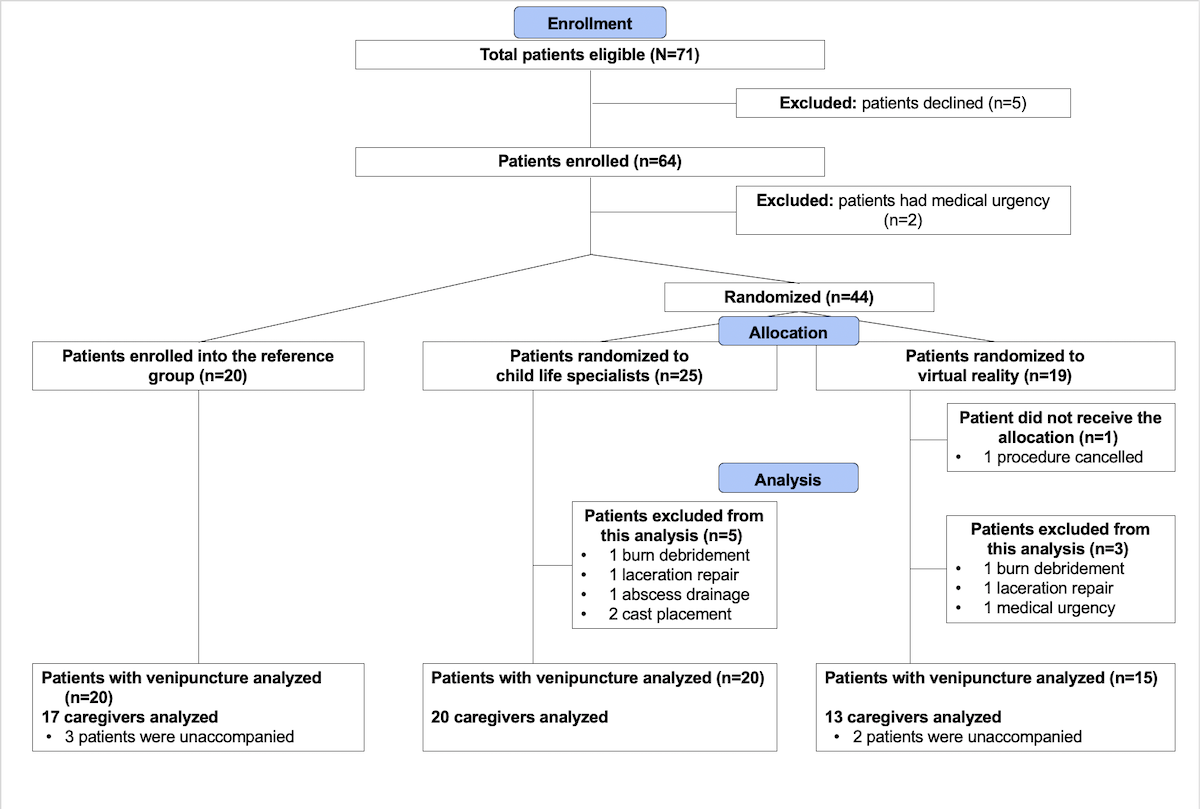
CONSORT patient flow diagram.

The eligibility, enrollment, and randomization procedures are shown in Figure 1. Recruitment occurred from June 2019 to March 2020 and was terminated owing to safety precautions during the COVID-19 pandemic.

### Feasibility

Out of the 71 patients invited to participate, 66 (93%) were recruited and 5 (7%) declined to enroll. Of the 15 patients in this study population who were randomized to VR, 4 patients (27%) withdrew from using VR. Patients who withdrew had issues related to the fit of the headset or distress with a preference to watch the procedure (Table 1). All completed procedures (63/63) had complete documentation of the parent and caregiver coping/distress behaviors.

**Table 1:** Characteristics of the patients who withdrew from the use of virtual reality during pediatric procedures.

Age (years)	Sex	Reason for virtual reality withdrawal	CAMPIS-SF^a^ scores (range 0-1.00)		Visual analog scale (range 0-10)		Cybersickness symptoms
			Patient coping	Patient distress	Peak pain	Peak anxiety	
10	Female	Declined because of poor headset fit and sliding down too much, virtual reality withdrawn before the procedure	1.00	0.00	7	1	No
10	Female	Distressed during the procedure, removed virtual reality to watch the procedure, withdrew in the middle of the procedure	0.46	0.54	5	9	No
8	Female	Patient rolling and flailing, virtual reality removed for safety and because the child preferred to see the procedure, withdrew in the middle of the procedure	0.43	0.57	10	10	No
7	Female	The patient was moving, virtual reality headset was slipping, so virtual reality removed at child life specialists’ and patient discretion, withdrawn near the end of the procedure.	0.89	0.11	6	3	No

^a^CAMPIS-SF: Child Adult Medical Procedure Interaction Scale-Short Form.

### Clinical Outcomes

A total of 55 patients undergoing venipuncture comprised the analysis of the clinical outcomes: 15 patients (15 caregivers) were randomized to VR group, 20 patients (15 caregivers) were randomized to child life specialist group, and 20 patients (17 caregivers) were included in the reference group. The mean age of all the patients was 14.1 (SD 4.1) years. Patient demographics were well-balanced across groups (Table 2). The mean procedure duration in minutes was 5.1 (SD 5.0), 7.7 (SD 5.5), and 11.6 (SD 7.0) for the reference, child life specialist, and VR groups, respectively. Analysis of the venipuncture procedural resources (eg, needle success rate) are reported in a secondary analysis [18]. Topical treatments (either lidocaine cream or cold spray) were applied to 0, 2, and 2 patients in the VR, child life specialist, and reference groups, respectively. Opiates (fentanyl, oxycodone, or morphine) were administered to treat pain in 0, 2, and 1 patients in the VR, child life specialist, and reference groups, respectively.

Patient coping differed across groups with higher coping in VR group and child life specialist group than in the reference group (*P*=.05). There were no significant differences in distress and pain ratings for patients and caregivers between the groups. Caregivers’ perception of their child’s anxiety also differed with the lowest perceived anxiety in the child life specialist group (*P*=.03). There was no change in cybersickness symptoms before and after VR use (*P*=.37) (Table 3).

**Table 2:** Patient demographics for clinical outcomes.

Patient demographics		Reference group	Child life specialist group	Virtual reality group	Total patients	*P* value	
Patients, n (%)		20 (36)	20 (36)	15 (27)	55 (100)	N/A^a^	
Age (years), mean (SD)		14.5 (4.2)	15.2 (4.0)	12.1 (3.5)	14.1 (4.1)	.08	
**Age category (years), n (%)**							.19
	Child (age range 7-9 years)	4 (20)	1 (5)	3 (20)	9 (15)		
	Early adolescent (age range 10-13 years)	3 (15)	7 (35)	6 (40)	19 (31)		
	Middle adolescent (age range 14-17 years)	9 (45)	5 (25)	5 (33)	22 (35)		
	Late adolescent/adult (age ≥18+ years)	4 (20)	7 (35)	1 (7)	12 (19)		
**Sex, n (%)**							.72
	Female	12 (60)	12 (60)	11 (73)	35 (64)		
	Male	8 (40)	8 (40)	4 (27)	20 (36)		
**Race, n (%)**							.20
	Black or African American	11 (55)	11 (55)	4 (27)	26 (47)		
	White	8 (40)	7 (35)	9 (60)	24 (44)		
	Unknown or not reported	1 (5)	2 (10)	2 (13)	5 (9)		
**Ethnicity, n (%)**							.30
	Hispanic or Latino	0 (0)	2 (10)	2 (13)	4 (7)		
	Not Hispanic or Latino	20 (100)	18 (90)	13 (87)	51 (93)		

^a^N/A: not applicable.

**Table 3:** Clinical outcomes of the patients and caregivers.^a^

Outcome				Reference group	Child life specialist group	Virtual reality group	*P* value	
**Patient outcomes**								
		Patients (N=55), n (%)		20 (36)	20 (36)	15 (27)	N/A^b^	
		**Child Adult Medical Procedure Interaction Scale score, mean (SD)**						
			Patient coping score	0.70 (0.39)	0.90 (0.14)	0.88 (0.19)	.046^c^	
			Patient distress score	0.20 (0.31)	0.10 (0.14)	0.12 (0.19)	.36	
		**Change in pain and anxiety scores, mean (SD)**						
			Pain	0.95 (2.35)	–1.20 (4.16)	–0.20 (4.31)	.19	
			Anxiety	1.45 (3.32)	–0.10 (1.74)	0.53 (2.77)	.20	
		Topical anesthetic used, n (%)		1 (5)	2 (10)	2 (13)	.21	
		**Cybersickness symptoms in children, n (%)**						.37
			Before virtual reality use	N/A	N/A	3 (20)		
			After virtual reality use	N/A	N/A	3 (20)		
**Caregiver outcomes**								
	Caregivers, n (%)			17 (36)	15 (32)	15 (32)	N/A	
	**Child Adult Medical Procedure Interaction Scale score, mean (SD)**							
			Caregiver coping promoting score	0.57 (0.43)	0.52 (0.48)	0.63 (0.38)	.76	
			Caregiver distress promoting score	0.20 (0.31)	0.08 (0.20)	0.24 (0.30)	.28	
	**Change in pain and anxiety scores, mean (SD)**							
			Caregiver’s perception of patient’s pain	0.00 (2.52)	–2.47 (3.50)	–1.73 (3.49)	.09	
			Caregiver’s perception of patient’s anxiety	1.29 (2.47)	–1.60 (2.64)	–0.53 (3.85)	.03^c^	
			Caregiver’s own anxiety	0.35 (1.32)	–0.73 (2.22)	–0.13 (3.09)	.41	

^a^Change in pain and anxiety scores ranges from –10 to 10. A negative value signifies reduced pain or anxiety during the procedure.

^b^N/A: not applicable.

^c^Significant at *P*<.05.

## Discussion

### Principal Findings

This study found that real-time objective behavior observations of patient and caregiver coping were feasible to perform in a study of VR use in the pediatric emergency department. The addition of objective behavioral observations in this study is a novel addition within the VR literature and may provide a complementary endpoint for future VR studies. Observations of patients’ behavior during medical procedures offer rich objective data that can support past studies on the effectiveness of VR on pain and anxiety [2,5,6,19]. Although real-time observations were feasible in this protocol, patient recruitment was slower than expected, in part due to patients who declined to enroll and research assistant availability. Future protocols may address this issue with augmented research staffing (eg, increased numbers of hours per day or days per week of active enrollment or increased numbers of research personnel) to maximize recruitment during the study period. The clinical outcomes offer preliminary data into the effect of VR on children’s coping during venipuncture. Patients in the VR and child life specialist groups exhibited similar coping during venipuncture, and both had higher coping than the group with no standardized support (reference group). While past findings show conflicting data on the effect of VR on pain and anxiety during pediatric procedures [1,6,19,20], our study suggests that VR and child life specialists both improve children’s coping during venipuncture. A study designed with coping as a primary endpoint is warranted to fully explore the link between coping and VR use.

Distraction is a psychological intervention that is effective at reducing pain in children undergoing needle-based interventions [21]. The immersive nature of VR makes it a deepened mode of distraction [22], and therefore, a possible modality to improve the experience of pediatric venipuncture. Given the recent physical distancing guidelines, alternate protocols can be explored whereby the staff can set up the VR headset remotely. A remote setup protocol for VR has considerable potential as a relatively hands-off and well-tolerated distraction tool. However, as caregivers perceived the lowest anxiety in the child life specialist group, the psychological benefit of a formal child life specialist support is evident.

Our protocol offered VR to children as young as 7 years. Other study protocols that used standard-sized VR headsets included children as young as 7-10 years [1-3,5,19,23]. In addition, unique to our protocol was the study of an off-the-shelf headset with VR games that were not specifically designed for child use during medical procedures. Our study found fairly low recruitment of children younger than 13 years, in part owing to a lack of children undergoing eligible procedures during periods of enrollment. Of the 9 children younger than 13 years who were assigned to VR, 4 removed the mask due to distress or poor fit. One consideration for inclusion criteria with VR use in a young school-aged child is the measurement of the head circumference or interpupillary distance. Proper VR headset fit may be better predicted by a patient’s head size than age alone. The efficacy of VR is influenced not only by its role as a distraction and immersion tool but also by the fit of the headset, maturity of the child, and their inherent ability to regulate their emotional state in an immersed environment. Effectiveness of a standard-sized VR headset and nonmedical games warrant deeper exploration to understand what key factors influence successful use of VR in children of different ages.

Another novel aspect of this protocol was the observation of caregiver behaviors during pediatric VR use. Caregivers’ comments and actions (eg, reassuring comments, apology, or empathetic statements) are well described antecedents of children’s distress [7,12,24]. VR as an immersive experience may reduce caregiver distressing behaviors, which may be, in part, due to the lack of need to overreassure a child who is distracted or due to the inability to observe facial expressions under a VR headset. Future studies may explore whether VR affects the child-caregiver dynamics, which may elucidate a new or evolving state of child-caregiver interactions during medical procedures.

### Limitations

This pilot study has several limitations. First, as this was a pilot study, we were not powered to detect clinically meaningful differences in several patient outcomes. Furthermore, owing to the nature of the study interventions, blinding was not possible for patients or study personnel. Thus, the effect of the novelty of VR or biases through informed consent may have influenced the clinical outcomes (eg, objective coping/distress or subjective self-reported pain/anxiety). Nevertheless, we have demonstrated that the collection of observational measures during VR is feasible and results obtained from this study provide important preliminary data for the design of larger interventional investigations. Next, owing to pediatric emergency department procedures, child life specialists were present to support children during VR use. Therefore, it is not possible to separate the effect of child life specialists from VR, and this is of particular concern for the patients who discontinued VR. This can also limit generalizability to clinical sites that use VR without child life specialists present. Of note, the procedures were not video recorded for later reviews and accordingly, intraobserver reliability of the evaluators was not calculated after their training period. For a future large-scale study using CAMPIS-SF, recordings of the procedures could be included in the protocol to ensure evaluator consistency. Finally, we found that an individual who was not performing the venipuncture procedure was needed to support VR use. This may have broader implications for scalability of VR use as child life specialists assisted patients with the fitting of the VR headset, navigation of menus or games, helped remove the headset urgently when it was not tolerated, and observed for cybersickness symptoms.

### Conclusion

The findings of this study demonstrate that real-time documentation of observed behaviors in pediatric patients and caregivers is feasible in a study protocol evaluating VR during medical procedures, particularly with sufficient research staff for recruitment. Better coping was observed in children receiving VR or child life specialist support during venipuncture procedures. Further studies including children/early adolescents is warranted to fully evaluate the benefits of VR on pediatric coping and on the child-caregiver dynamics.

## Appendix

Multimedia Appendix 1 Child adult medical procedure interaction scale-short form codes.

Multimedia Appendix 2 CONSORT-EHEALTH checklist (V. 1.6.1).

## Abbreviations

CAMPIS-SF: Child Adult Medical Procedure Interaction Scale-Short Form
VR: virtual reality
