# Supplementary figures and images for: Pediatric Coping During Venipuncture With Virtual Reality: Pilot Randomized Controlled Trial
Source: JMIR Pediatr Parent. 2021 Jul 28;4(3):e26040. doi: 10.2196/26040 (PMC8367183; doi:10.2196/26040)

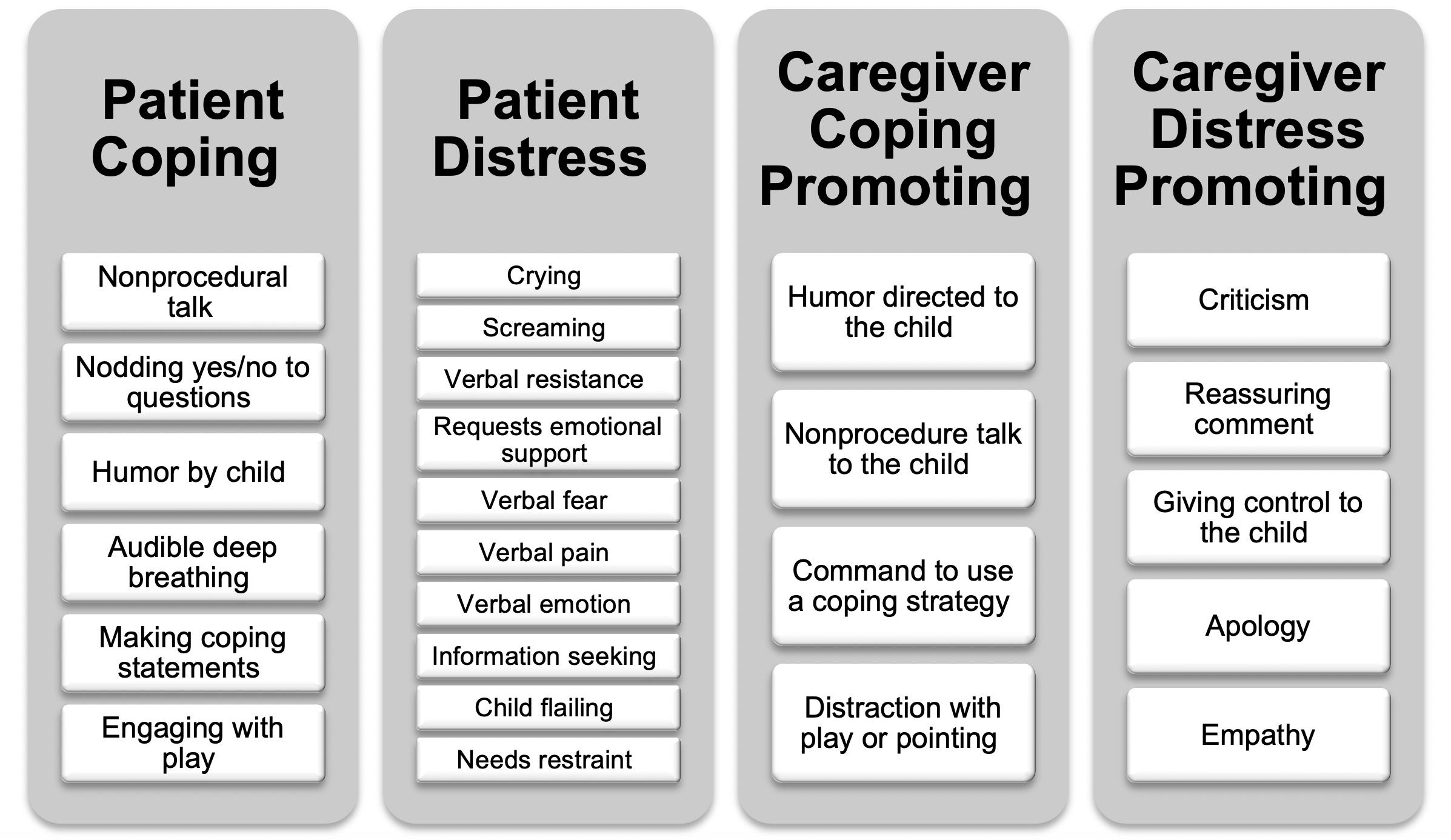

Supplement: Multimedia Appendix 1 [file pediatrics_v4i3e26040_app1.png]
